# Efficient end-to-end long-read sequence mapping using minimap2-fpga integrated with hardware accelerated chaining

**DOI:** 10.1038/s41598-023-47354-8

**Published:** 2023-11-17

**Authors:** Kisaru Liyanage, Hiruna Samarakoon, Sri Parameswaran, Hasindu Gamaarachchi

**Affiliations:** 1 School of Computer Science and Engineering, University of New South Wales, Sydney, NSW Australia; 2 Genomics Pillar, Garvan Institute of Medical Research, Sydney, NSW Australia; 3 Centre for Population Genomics, Garvan Institute of Medical Research and Murdoch Children’s Research Institute, Melbourne, Australia; 4 School of Electrical and Information Engineering, University of Sydney, Sydney, NSW Australia

**Keywords:** Computational biology and bioinformatics, Software, Electrical and electronic engineering

## Abstract

*minimap2* is the gold-standard software for reference-based sequence mapping in third-generation long-read sequencing. While *minimap2* is relatively fast, further speedup is desirable, especially when processing a multitude of large datasets. In this work, we present *minimap2-fpga*, a hardware-accelerated version of *minimap2* that speeds up the mapping process by integrating an FPGA kernel optimised for chaining. Integrating the FPGA kernel into *minimap2* posed significant challenges that we solved by accurately predicting the processing time on hardware while considering data transfer overheads, mitigating hardware scheduling overheads in a multi-threaded environment, and optimizing memory management for processing large realistic datasets. We demonstrate speed-ups in end-to-end run-time for data from both Oxford Nanopore Technologies (ONT) and Pacific Biosciences (PacBio). *minimap2-fpga* is up to 79% and 53% faster than *minimap2* for $\sim 30\times$ ONT and $\sim 50\times$ PacBio datasets respectively, when mapping without base-level alignment. When mapping with base-level alignment, *minimap2-fpga* is up to 62% and 10% faster than *minimap2* for $\sim 30\times$ ONT and $\sim 50\times$ PacBio datasets respectively. The accuracy is near-identical to that of original *minimap2* for both ONT and PacBio data, when mapping both with and without base-level alignment. *minimap2-fpga* is supported on Intel FPGA-based systems (evaluations performed on an on-premise system) and Xilinx FPGA-based systems (evaluations performed on a cloud system). We also provide a well-documented library for the FPGA-accelerated chaining kernel to be used by future researchers developing sequence alignment software with limited hardware background.

## Introduction

Third-generation long-read sequencing has gained significant popularity in the last few years and was recently acclaimed as the Method of the Year 2022^1^. In numerous long-read sequence analysis pipelines, *minimap2*^2^ is used as the gold standard tool for sequence mapping/alignment. Notably, *minimap2*^2^ is the preferred choice for sequence mapping in pipelines offered by leading third-generation sequencing companies, such as ONT (Guppy aligner) and PacBio (pbmm2). Due to *minimap2*’s ubiquitous use, several previous studies have attempted to accelerate *minimap2*^3–6^. However, most of these efforts have focused on optimising isolated parts of *minimap2* in a quarantined environment (not end-to-end integrated), with the only exception being CPU-based optimisations (using Intel AVX-512 instructions) by Kalikar et al.^3^.

End-to-end integration of optimisations back into bioinformatics software is crucial to harness the full potential of these optimisations in a practical and realistic manner. In fact, only a complete end-to-end integration can warrant that any optimisation results in a truly useful and efficient bioinformatics workflow. Integrated accelerations ensure that the performance gains in individual components seamlessly translate into overall improvements in the software run-time, ultimately expediting the processing and enhancing the efficiency of bioinformatics workflows. In this paper, we present *minimap2-fpga*, a Field Programmable Gate Array (FPGA) based hardware-accelerated version of *minimap2* that is end-to-end integrated. *minimap2-fpga* speeds up the mapping process of the widely used and routinely performed bioinformatics workflow - reference-based sequence mapping. *minimap2-fpga* supports mapping reads from both ONT and PacBio (though giving a higher speed-up for ONT read mapping).

The acceleration of the chaining step within *minimap2* has been explored in numerous previous studies, given its status as a key computational bottleneck within the software^3–6^. Sadasivan et al.^4^ used GPUs for accelerating this chaining step. Guo et al.^5^ accelerated the chaining step using FPGA, mainly targeting the read overlap mapping functionality in *minimap2*. Our previous work^6^ accelerated chaining using FPGAs targeting human reference-based mapping functionality in *minimap2*. These three works that use loosely coupled accelerator platforms (GPUs, FPGAs), only accelerate chaining in isolation and end-to-end integration is not performed with *minimap2*, thus limiting their practical utility. In contrast, Kalikar et al.^3^ use tightly coupled AVX-512 vector extensions available in modern Intel CPUs to accelerate chaining and integrate the acceleration to *minimap2*.

The isolated chaining step accelerator in our previous work^6^ combined a pipelined and parallel FPGA-based hardware architecture with a multi-threaded software execution of the chaining step computation. The system was able to split chaining tasks between CPU (multi-threaded) and FPGA (multi-kernel) platforms efficiently and process them in parallel to speed up the computation. Moreover, we demonstrated its capability to effectively process large-scale and realistic datasets frequently encountered in bioinformatics workflows. In this work, we integrate our previous FPGA-based hardware architecture^6^ into *minimap2* to enable end-to-end program execution, while exposing the accelerated chaining kernel as a well-documented software library.

Integrating the FPGA kernel into *minimap2* posed significant challenges when compared to designing the FPGA core presented in our prior work^6^. While data transfer overhead was previously considered^6^, the integration process exposed additional scheduling overheads when working with the multi-threaded execution environment in *minimap2*, resulting in marginal end-to-end speedup. In this paper, we present optimisation techniques for the splitting of chaining computations between the hardware accelerator and the CPU, while efficiently scheduling hardware-chosen computations on the accelerator, to achieve further acceleration.

We also discuss an unexpected caveat that arose when integrating the design, impacting final mapping accuracy, a factor not apparent in isolated testing environments. We detail how the FPGA design was modified to mitigate this issue. We believe that our findings will prove valuable for other bioinformatics tools requiring heterogeneous computing accelerations, emphasising the importance of integrating accelerated kernels back into software workflows. Our work highlights the potential for disruptive challenges which arise when integrating isolated acceleration of a single algorithm in a complex software environment, underpinning the need for end-to-end system integration.

Our end-to-end integrated *minimap2-fpga* requires a heterogeneous computing system that contains a CPU connected to an FPGA through PCI-Express. FPGA from the mainstream FPGA vendors Xilinx (now under AMD) and Intel (former Altera) are supported. Further, we have ensured that our implementation works on both locally set up (on-premises) computing systems and the systems available on the cloud (Amazon AWS and Intel DevCloud). *minimap2-fpga* is available at https://github.com/kisarur/minimap2-fpga and the accelerated chaining library called *chain-fpga* is available at https://github.com/kisarur/chain-fpga.

## Results

### *minimap2-fpga* and *chain-fpga*

*minimap2-fpga* is an FPGA-accelerated version of the *minimap2* software designed for reference-based sequence mapping. *minimap2-fpga* can be run using the same commands as the original *minimap2* software. The repository has separate branches for different FPGA platforms. The *xilinx* branch of the repository contains the source code for Xilinx FPGA, while the *intel* branch contains the source code for Intel FPGA. Despite being developed using platform-agnostic OpenCL, the build workflows and HLS pragmas used to generate efficient hardware differed between the two platforms, resulting in the need for separate branches. To enhance user convenience, we provide binaries for two platforms: 1. Intel Arria 10GX FPGA and 2. Xilinx UltraScale+ VU9P based FPGA available on cloud AWS EC2 F1 instances. An example of how a user can set up and execute *minimap2-fpga* on an AWS cloud (*f1.2xlarge* instance loaded with *FPGA Developer AMI Version 1.12.1*) is as below:
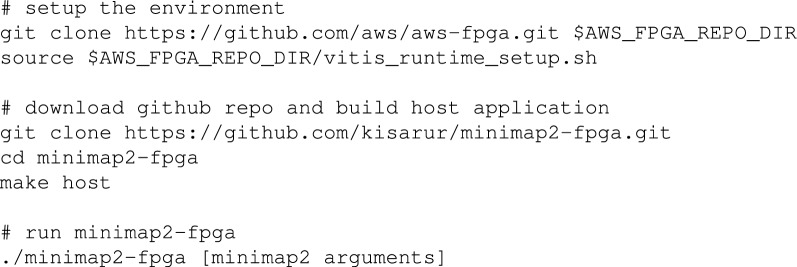


*chain-fpga* is the isolated library API for the FPGA-based chaining which could be used in various bioinformatics tool development where chaining is a critical computational hotspot (e.g., sigmap^7^). This library API offers an easy-to-use interface for integrating the chaining accelerator into other bioinformatics tools without requiring expertise in custom hardware development. Similar to the *minimap2-fpga* repository, this repository also contains two separate branches for Xilinx and Intel FPGA, along with pre-compiled binaries. The following are the three key API functions supported by the library API:
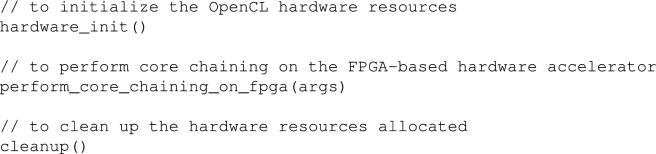


### Performance comparison

The end-to-end run time of *minimap2-fpga* was benchmarked on a cloud Xilinx FPGA system and an on-premises Intel FPGA system. To represent realistic workloads, two large and complete datasets were employed for the benchmarks: a $\sim 30\times$ ONT human dataset with a size of 176 GB and a $\sim 50\times$ PacBio CCS human dataset with a size of 311 GB (see Materials and Methods). This intentional selection distinguishes our work from previous hardware-accelerated systems, which only performed benchmarks on small datasets , possibly due to challenges in managing memory effectively. For example, FPGA based *minimap2*’s chaining step acceleration by Guo et al.^5^ has been benchmarked against a 4.6 GB sized Caenorhabditis Elegans dataset and as we have shown in our prior work^6^, the maximum human dataset size it could support was 32 GB. We compared the run time of *minimap2-fpga* with the original *minimap2* software in two different modes for each system and dataset: one with base-level alignment enabled (w/alignment) and the other without (w/o alignment).

**Figure 1:**
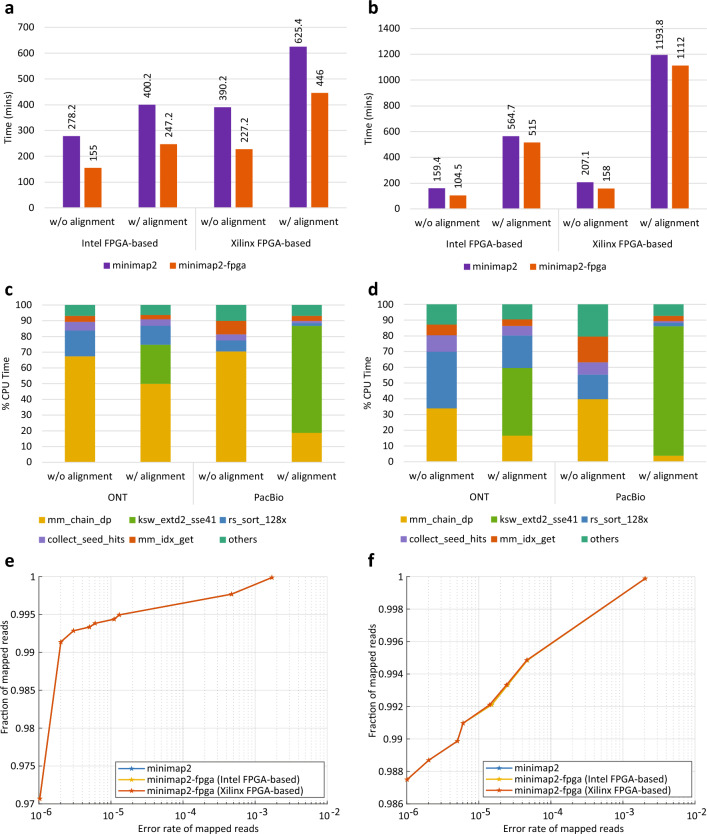
Run-time performance, function-level profiling and accuracy comparisons of *minimap2* vs. *minimap2-fpga*. (**a**) Run-time performance results of original *minimap2* vs. *minimap2-fpga* for ONT $\sim 30\times$ dataset. (**b**) Run-time performance results of original *minimap2* vs. *minimap2-fpga* for PacBio $\sim 50\times$ dataset. (**c**) Function-level profiling results of original *minimap2*. Profiling was done with the Perf profiler^8^ on Intel FPGA-based system for subsets of ONT and PacBio datasets. (**d**) Function-level profiling results of *minimap2-fpga*. Profiling was done with the Perf profiler^8^ on Intel FPGA-based system for subsets of ONT and PacBio datasets. (**e**) Accuracy comparison performed with simulated ONT reads for original *minimap2* vs. *minimap2-fpga* with base-level alignment (raw values in Table 1, plotted data points in Supplementary Table S1). (**f**) Accuracy comparison performed with simulated ONT reads for original *minimap2* vs. *minimap2-fpga* without base-level alignment (raw values in Table 1, plotted data points in Supplementary Table S1).

For nanopore data (Fig. 1a), *minimap2-fpga* is 79% faster than *minimap2* on the on-premise Intel FPGA system and 72% faster than *minimap2* on the cloud Xilinx FPGA system when mapping without base-level alignment. When mapping with base-level alignment enabled, *minimap2-fpga* shows a speedup of 62% and 40% on the on-premise and cloud systems, respectively. On PacBio data (Fig. 1b), *minimap2-fpga* is 53% faster than *minimap2* on the on-premise Intel FPGA-based system and 31% faster on the cloud Xilinx FPGA-based system when mapping without base-level alignment. With base-level alignment enabled, *minimap2-fpga* shows a speedup of 10% and 7% on the on-premise Intel and cloud Xilinx systems, respectively. It should be noted that these speed-ups represent end-to-end run-time improvements of *minimap2-fpga* over *minimap2* under multi-threaded executions. *minimap2-fpga*’s end-to-end run-time includes all overheads due to the hardware accelerator (e.g., initialisation, data transfer between the host and the device, hardware scheduling, etc.), in addition to all steps from reading the genomic reads from the disk to writing the alignment results to the disk.

The variation in performance improvement across different datasets and modes can be attributed to the proportion of time spent on chaining (i.e. “mm_chain_dp” function which is the FPGA-accelerated component), out of the total execution time (Fig. 1c). For nanopore data, original *minimap2* spends 67% of its time on chaining when performed without base-level alignment (Fig. 1c), and this is reduced to 34% after FPGA acceleration (Fig. 1d). When nanopore data is processed with base-level alignment, the chaining component on original *minimap2* takes up 50% of the total time, but this drops to 17% after being accelerated on FPGA. For PacBio data, the original *minimap2* spends 71% of its total execution time on chaining when mapping without base-level alignment, which is reduced to 40% after FPGA acceleration. When PacBio data is processed with base-level alignment, *minimap2* spends only 19% of its total execution time on chaining, which is reduced to 4% after being accelerated on FPGA.

### Accuracy evaluation

The accuracy evaluation of the final mappings was performed using two methods; 1. reference software (*minimap2*) independent method that uses simulated reads where the exact mapping location is known; 2. reference software (*minimap2*) dependent method that considers real reads mapped using original *minimap2* to be the truth set.

#### Using simulated reads

**Table 1:** Accuracy comparison with ONT simulated reads.Significant values are in bold.

MAPQ_T	*minimap2*		*minimap2-fpga*			
			Intel FPGA-based system		Xilinx FPGA-based system	
	No. reads with MAPQ > $=$ MAPQ_T	No. wrong mappings	No. reads with MAPQ > $=$MAPQ_T	No. wrong mappings	No. reads with MAPQ > $=$ MAPQ_T	No. wrong mappings
With base-level alignment						
60	**960462**	0	**960470**	0	**960464**	0
59	**10210**	1	**10213**	1	**10209**	1
38	**20705**	1	**20694**	1	**20704**	1
8	1477	1	1477	1	1477	1
5	492	2	492	2	492	2
4	492	1	492	1	492	1
3	**546**	5	**544**	5	**544**	5
2	**568**	2	**570**	2	**570**	2
1	2730	456	2730	456	2730	456
0	2198	1241	2198	1241	2198	1241
Unmapped	120		120		120	
Without base-level alignment						
60	**970514**	0	**970515**	0	**970516**	0
22	**16981**	1	16981	1	**16979**	1
6	**1193**	1	**1194**	1	**1195**	1
5	**1176**	3	**1177**	3	**1177**	3
4	**1107**	1	**1106**	1	**1104**	1
3	**1131**	9	**1130**	9	**1130**	8
2	**1223**	10	**1222**	10	**1222**	10
1	1525	22	1525	22	1525	22
0	5030	1985	5030	1985	5032	1985

Differences are highlighted in bold.

For nanopore reads, the accuracy of *minimap2-fpga* is near-identical to the accuracy of original *minimap2* for both with base-level alignment (Fig. 1e) and without base-level alignment (Fig. 1f), on both computer systems (Intel FPGA and Xilinx FPGA systems), as demonstrated by the accuracy curves overlapping one another. The minuscule differences in the accuracy are highlighted in bold in Table 1. The top half of Table 1 contains the accuracy evaluation for sequence mapping with base-level alignment enabled and the bottom half is for sequence mapping without base-level alignment. In Table 1, the first column shows the mapping quality threshold (MAPQ_T). For each system in Table 1, the corresponding first column gives the number of reads mapped with MAPQ>=MAPQ_T and the second column gives the number of wrong mappings included in those mappings. Based on data in Table 1, error rate of mapped reads (accumulative number of wrong mappings/accumulative number of mapped reads) and the fraction of mapped reads (accumulative number of mapped reads/total number of mapped reads) were calculated (Supplementary Table S1) and plotted (Fig. 1e and f). In Fig. 1e and f, the horizontal axis show the error rate of mapped reads and the vertical axis show the fraction of mapped reads corresponding to the error rate.

**Table 2:** Accuracy comparison with PacBio simulated reads.

MAPQ_T	*minimap2*		*minimap2-fpga*			
			Intel FPGA-based system		Xilinx FPGA-based system	
	No. reads with MAPQ > $=$ MAPQ_T	No. wrong mappings	No. reads with MAPQ > $=$ MAPQ_T	No. wrong mappings	No. reads with MAPQ > $=$MAPQ_T	No. wrong mappings
With base-level alignment						
60	**1167692**	0	**1167691**	0	**1167691**	0
1	**1499**	70	**1500**	70	**1500**	70
0	961	549	961	549	961	549
Unmapped	64		64		64	
Without base-level alignment						
60	**1164914**	0	**1166460**	0	**1166460**	0
1	**1549**	**1**				
0	**3738**	**795**	**3741**	**793**	**3741**	**792**

Differences are highlighted in bold.

Similar to nanopore reads, the accuracy for PacBio reads from *minimap2-fpga* is also near-identical to that of original *minimap2* (Table 2, which has the same structure as Table 1 explained in the previous paragraph). The mapping quality (MAPQ) score distribution for PacBio reads had only 3 distinct values for both with and without base-level alignment. For both with and without base-level alignment, the number of reads mapped with MAPQ>=MAPQ_T by *minimap2-fpga*, is equal to or even slightly higher than that of *minimap2*. The number of unmapped reads was the same on both *minimap2* and *minimap2-fpga*.

#### Using real reads

For the $\sim 30\times$ ONT dataset, 23,228 out of 9,083,052 reads were unmapped in both versions of *minimap2* (*minimap2* and *minimap2-fpga*) on both Intel FPGA-based system and the Xilinx FPGA-based system (Table 3). On the Intel FPGA-based system, 9,045,287 mappings were the same in both versions of *minimap2*, making the output similarity between the two versions 99.58%. On the Xilinx FPGA-based system, 9,053,718 mappings were the same in both versions of *minimap2*, making the output similarity between the two versions 99.68%.

**Table 3:** Accuracy comparison with real reads.

	Intel FPGA-based System		Xilinx FPGA-based System	
	ONT	PacBio	ONT	PacBio
Total reads	90,83,052	1,17,14,594	90,83,052	1,17,14,594
Unmapped in both	23,228	62,461	23,228	62,492
Same	90,45,287	1,16,25,209	90,53,718	1,16,35,943
Mismatches	14,535	26,883	6106	16,159
Exclusively aligned by *minimap2*	2	10	0	0
Exclusively aligned by *minimap2-fpga*	0	31	0	0
Same %	99.58	99.24	99.68	99.33

For the $\sim 50\times$ PacBio dataset, 62,461 and 62,492 out of 11,714,594 reads were unmapped in both versions of *minimap2* on Intel FPGA-based system and the Xilinx FPGA-based system, respectively. On the Intel FPGA-based system, 11,625,209 mappings were the same in both versions of *minimap2*, making the output similarity between the two versions 99.24%. On the Xilinx FPGA-based system, 11,635,943 mappings were the same in both versions of *minimap2*, making the output similarity between the two versions 99.33%.

The output similarity between *minimap2* and *minimap2-fpga* is higher on the Xilinx FPGA-based system than on the Intel FPGA-based system due to the higher maximum loop trip count (H=1024 vs H=512) possible on Xilinx FPGA-based system (detailed in Materials and Methods).

### Energy-delay product comparison

To evaluate the energy efficiency of our proposed work, we estimated the energy-delay product (Materials and Methods, Supplementary Note 5) of both *minimap2* and *minimap2-fpga* on the cloud Xilinx FPGA-based system using the $\sim 30\times$ ONT and $\sim 50\times$ PacBio datasets. For the ONT dataset, *minimap2-fpga* demonstrates a 67.13% reduction in the energy-delay product compared to *minimap2* when mapping without base-level alignment; and, 46.55% when mapping with base-level alignment. For the PacBio dataset, these values were 54.85% and 5.53%, respectively.

## Discussion

### Previous acceleration work

Second-generation sequence analysis has been extensively optimized through various approaches, including CPU-based optimizations^9,10^, GPU-based optimizations^11^ and FPGA-based optimizations^12,13^ and custom MPSoC designs^14^. FPGA-based accelerations include a work^12^ that achieves a speedup over second-generation sequence analysis tool - Bowtie^15^ in both single-core and 16-core performance, a work^16^ that accelerates BWA-SW aligner^17^, and an end-to-end integrated tool^13^ with output accuracy comparable to BWA-MEM^18^. The survey by Ng et al.^19^ assesses the performance of FPGA-based acceleration work for second-generation sequence analysis under four main categories: pairwise sequence alignment, database search, multiple sequence alignment, and mapping. The survey by Salamat and Rosing^20^ explores the FPGA-based accelerations of various DNA alignment algorithms, including Smith-Waterman, hash-table-based algorithms, and BWT with FM-index, among others. Commercial FPGA-based solutions, such as the DRAGEN system accompanying Illumina sequencers, have also emerged, benefiting from prior academic works in this field.

The emergence of third-generation sequencing, which offers massive throughput and ultra-long reads, has amplified the need for accelerated sequence analysis. CPU-based optimizations for third-generation sequence analysis include the acceleration of base-level alignment of *minimap2* on three processors^21^, multi-stage acceleration of *minimap2* on modern CPUs with Intel AVX-512 instructions^3^. GPU-based optimizations include the acceleration of adaptive banded event alignment in ONT raw signal analysis^22^ and acceleration of chaining step of *minimap2*^4^. FPGA-based solutions include acceleration of the base-calling task in Oxford Nanopore sequence analysis^23^, an integration^24^ of the GACT-X aligner architecture^25^ with *minimap2*, acceleration of *minimap2*’s chaining step^5,6^ and acceleration of selective genome sequencing^26^.

### Challenges in hardware-software integration

Accelerating an isolated section of a large software tool on hardware accelerator platforms is commonly seen in the domain of hardware acceleration. However, integrating such accelerators with the rest of the software tools is less common and can pose significant challenges. A naive integration could diminish the astounding speed-up gained by the accelerator alone for the isolated portion of the software tool, and in some cases, even slow it down. Overcoming these challenges is necessary to achieve a speed-up in the end-to-end time. However, in the bioinformatics domain, integrating such accelerators with the software tool is crucial to provide a complete genomics analysis flow that can run with the accelerated tool.

*minimap2-fpga* is an end-to-end FPGA accelerated version for the gold-standard third-generation genomics sequence alignment tool *minimap2*. This integration resulted in a significant speed-up in the end-to-end time of the tool, while preserving the accuracy of the final output. Achieving this speed-up while maintaining accuracy required careful fine-tuning of all stages of the hardware-software processing pipeline, which involved addressing several engineering challenges. To enhance the accuracy of the final output while staying within the FPGA’s logic resource constraints, we made novel architectural changes to our previous hardware accelerator for isolated chaining^6^. We also created linear-regression based models to predict the time taken for each chaining task on hardware and software, allowing for more intelligent task-splitting decisions. To optimize access to a smaller number of hardware accelerator units from a larger number of software threads, we developed a scheduling mechanism.

### OpenCL for accelerating bioinformatics kernels

We utilized OpenCL High-Level Synthesis (HLS) instead of a Hardware Descriptive Language (HDL) such as Verilog or VHDL for hardware accelerator implementation. HLS allowed for the accelerator to be designed using a high-level language similar to the C programming language, with the HLS compiler being considerably guided manually by appropriate compiler directives to generate the desired hardware. While using HDL instead of HLS could have improved the performance and FPGA resource usage of the accelerator by allowing for fine-tuning and optimization at a finer granularity, using HLS provided greater flexibility in making changes to an already designed hardware with significantly less development time. For instance, when the chaining step accelerator in this work needed to be modified from the previous version^6^ to the current version to improve the accuracy of the output it generates, the changes could be implemented (after the architectural changes were decided at a higher level) with relatively lesser time and effort. If this were to be done with HDL, it would require significantly more time and effort to implement, verify and debug the additional features added to the accelerator. This is particularly beneficial for bioinformatics software like *minimap2*, which is fast-evolving and requires quick implementation of future algorithm changes in the hardware.

### Future work

Once the chaining step of *minimap2* is accelerated by $\sim 4\times$ (on average) in this work, the other sections of the tool such as *ksw_extd2_sse41*, *rs_sort_128x* have now become the hotspots (see Fig. 1d). To get even better improvements in the tool’s end-to-end time, future research can design hardware accelerators for these sections as well and integrate the accelerators into the rest of the software using the same techniques discussed in this work. In addition to creating specialized hardware, some work^3,27^ have already improved the software performance of core algorithms used in these remaining hotspots and such work can also be integrated into our work to get better performance improvements in end-to-end time. Furthermore, for sequence analysis applications such as selective sequencing, which sequences specific regions or targets of interest within the genome, *minimap2* is employed without base-level alignment (e.g. *readfish*^28^). *minimap2-fpga*, which offers higher speed-ups for mapping tasks that do not require base-level alignment, can be used as a replacement for *minimap2* to achieve speed-ups in such selective sequencing applications.

## Materials and methods

### Computer systems and datasets

To evaluate the performance and accuracy with real sequencing data, two publicly available datasets were used: a NA12878 sample sequenced on an ONT PromethION sequencer (SRA Accession no. SRX11368472, 9.1M reads, 93.4 Gigabases^29^); and an HG002/NA24385 sample sequenced on a PacBio Sequel II (15+20 kb CCS, SRA Accession no. PRJNA586863, 11.7M reads, 166.2 Gigabases^30^). To evaluate accuracy using simulated sequencing data: 1 million synthetic ONT long reads were simulated from hg38 human reference genome using *squigulator*^31^ (ONT raw signal data in BLOW5 format^32^) followed by *buttery-eel*^33^ (basecall signal data); and, $\sim 1.2$ million synthetic PacBio CCS reads were simulated using *pbsim*^34^ from hg38 reference.

The on-premise Intel FPGA-based system used for performance and accuracy benchmarking was a heterogeneous computing system comprised of a 20-core Intel Xeon Gold 6148 CPU (2.40 GHz) with 754 GB of RAM and an Intel Programmable Acceleration Card (Intel Arria 10 GX 1150 FPGA) with 8 GB DDR4 onboard memory. The cloud Xilinx FPGA-based system was an Amazon EC2 F1 instance (*f1.2xlarge*) with 8 vCPUs, 122 GiB instance memory and a Xilinx UltraScale+ VU9P based FPGA device.

### Performance, accuracy and energy-delay product evaluation

To compare the runtime performance of *minimap2* and *minimap2-fpga*, the $\sim 30\times$ ONT and the $\sim 50\times$ PacBio datasets were mapped against hg38 human reference genome using *minimap2-fpga* and *minimap2*, with and without base-level alignment. Both *minimap2* and *minimap2-fpga* were run with 8 CPU threads, on both cloud Xilinx FPGA system and on-premise Intel FPGA system (Supplementary Note 4).

To evaluate accuracy using simulated sequencing data, the generated simulated reads were mapped to the hg38 human reference genome using *minimap2-fpga* and *minimap2*, with and without base-level alignment. The outputs from both the computer systems were then evaluated using the *mapeval* utility in *paftools* scripts available under the *minimap2* repository. For real data (ONT $\sim 30\times$ and the PacBio $\sim 50\times$ datasets), the mapping output generated by original *minimap2* (with base-level alignment) software was considered the truth-set. The output generated by *minimap2-fpga* (with base-level alignment) for real data was compared against the truth-set using a SAM comparison utility^35^ (Supplementary Note 4).

To evaluate the energy efficiency of *minimap2-fpga* and *minimap2*, the energy-delay product ($EDP = Energy \times Runtime$ where $Energy = Power \times Runtime$) was estimated on the cloud Xilinx FPGA-based system, for both $\sim 30\times$ ONT and $\sim 50\times$ PacBio datasets (Supplementary Note 5). The FPGA power consumption was measured using the *fpga-describe-local-image -S 0 -M* command^36^ available on the Amazon EC2 F1 instances. Due to the unavailability of a dedicated measurement method for the Intel Xeon E5-2686 v4 CPU on the cloud Xilinx FPGA-based system, the CPU power consumption was estimated by scaling the thermal design power (TDP) by the CPU core utilisation of *minimap2-fpga* and *minimap2* (Supplementary Note 5). Note that the energy estimation was only conducted for the cloud Xilinx FPGA-based system, as we did not have root permission for the on-premise Intel FPGA-based system.

### *minimap2-fpga* development, optimisation and integration

**Figure 2:**
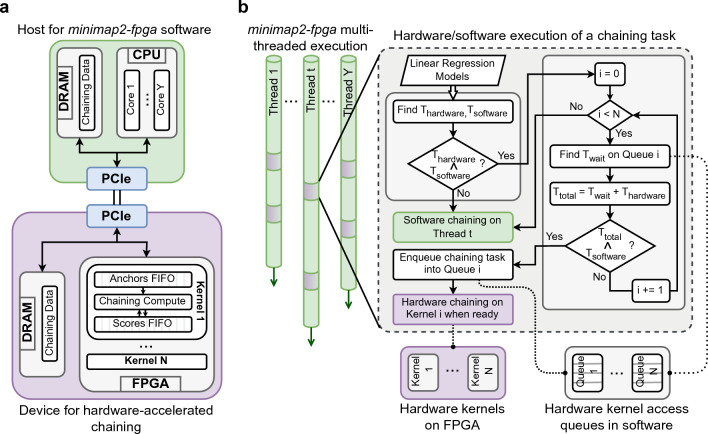
Overall system architecture and the execution flow of *minimap2-fpga* (**a**) Architecture of the CPU-FPGA heterogeneous system running *minimap2-fpga*. The multi-core CPU (Core 1, ..., Y), within the host, handles software executions, while the FPGA, within the device, hosts multiple hardware kernels (Kernel 1, ..., N) designed for hardware-accelerated chaining. They are connected via PCI Express for host-device communication. (**b**) Overall execution flow of hardware-software integrated *minimap2-fpga*. Multiple threads (upto Y) are launched by the CPU when *minimap2-fpga* starts. When a thread reaches the chaining stage, the chaining task is processed either on FPGA as hardware or on the same CPU thread as software, based on predicted execution and wait times. As a thread (denoted as *t*) reaches a point requiring the execution of a chaining task, it predicts the time needed for this task to complete on both hardware ($T_{hardware}$) and software ($T_{software}$). If $T_{software} \le T_{hardware}$, the chaining task is executed on the same thread *t* as software. If $T_{hardware} < T_{software}$, *minimap2-fpga* attempts to schedule the task on one of the *N* available hardware kernels, taking into account the wait time ($T_{wait}$) associated with accessing each hardware kernel. It searches for a kernel where the total processing time on hardware ($T_{total} = T_{wait} + T_{hardware}$) is less than $T_{software}$, and if such a kernel is found, the chaining task is queued into the hardware access queue specific to that kernel (queue *i*) and later processed on the corresponding hardware kernel (kernel *i*) once the kernel is ready. If not, the task proceeds as software execution on the same thread *t*. This prediction-based dynamic hardware scheduling approach optimizes the utilization of smaller number of hardware kernels (*N*) from a higher number of software threads (upto *Y*) while ensuring efficient multi-threaded performance.

*minimap2-fpga* was implemented using C/C++ and OpenCL framework. To accelerate the compute-intensive chaining step, *minimap2-fpga* utilizes either the custom FPGA-based hardware accelerator (implemented using OpenCL HLS, extending our previous work^6^) or the CPU-based software implementation, based on the predicted hardware/software execution times of each chaining task. To schedule chaining tasks on hardware while minimising the waiting times to access hardware accelerator kernels, an optimized scheduling algorithm has been introduced. Memory management in *minimap2-fpga* has been optimised for handling large realistic datasets in reference-based sequence mapping efficiently.

#### CPU-FPGA heterogeneous system architecture

The proposed hardware-software heterogeneous system running *minimap2-fpga* is a host-device architecture interconnected through a PCI Express (PCIe) interface (Fig. 2a). The host component contains a multi-core CPU, capable of running software with parallel threads. The device contains an FPGA, configured with multiple hardware kernels, each capable of processing a separate chaining task (a detailed view of a hardware kernel is available in Supplementary Fig. S2). Note that the number of hardware kernels that can be configured on the FPGA (*N*) is usually less than the number of cores available in a CPU (*Y*) of a typical high-performance computing (HPC) system. When a chaining task necessitates hardware processing, input/output data associated with the respective chaining task is transferred between the host’s DRAM and the device’s DRAM via the PCIe interface utilizing Direct Memory Access (DMA) techniques facilitated by the OpenCL framework.

#### Hardware accelerator development and optimisation

Intel Arria 10 GX FPGA Acceleration Stack 1.2.1 (with Intel FPGA SDK for OpenCL 19.4 and Intel Quartus Prime Pro 19.2) was used for hardware accelerator development on the on-premise Intel FPGA-based system (Supplementary Note 1). On the cloud Xilinx FPGA system, AWS EC2 FPGA Development Kit with Xilinx Vitis v2021.2 was used for the hardware accelerator development. For Xilinx FPGA, the OpenCL HLS pragmas used in the Intel FPGA hardware accelerator implementation had to be converted to supported Xilinx OpenCL HLS pragmas. Due to the lack of a pragma in Xilinx OpenCL HLS for data prefetching from global memory to local memory (lines 10-11 in Supplementary Algorithm 1), this prefetching was manually implemented.

We enhanced our previous hardware accelerator^6^, to achieve a level of accuracy in the final mapping output that is comparable to that from the original *minimap2* software. When we naively integrated our previous hardware accelerator^6^ to *minimap2*, the end-to-end accuracy was considerably lower in comparison to the original *minimap2* (Supplementary Fig. S1). Further investigation revealed that setting a maximum loop trip count in the chaining algorithm to 64 was the culprit (*H*=64 in line 17 of Supplementary Algorithm 2). To improve the accuracy, the maximum trip count had to be increased. However, naively increasing this trip count considerably increased the resource usage on the FPGA which resulted in a design that could not fit the available FPGA area (in terms of the number of logic components such as LUTs, FFs and DSPs). To improve accuracy while keeping the resource usage low, we restructured the affected inner loop into sub-partitions (detailed in Supplementary Note 1). With this improvement to the hardware accelerator, a maximum trip count of 512 (i.e. *H*=512) and 1024 (i.e. *H*=1024) were achieved on the Intel FPGA and the Xilinx FPGA accelerator, respectively. A single hardware kernel (i.e. *N*=1 in Supplementary Fig. S2) was configured on both the Intel and Xilinx FPGA accelerators. The overall accuracy is now near-identical to the original *minimap2* (Fig. 1e and f).

#### Memory management optimisation

To efficiently handle large realistic datasets commonly encountered in reference-based sequence mapping, the memory management in *minimap2-fpga* was carefully optimized. Rather than transferring the entire dataset to the limited FPGA device DRAM upfront (as done in prior FPGA-based related work^5^), we adopted a more efficient approach: transferring the data relevant to hardware-executed chaining task, task-by-task, just before the task’s execution. By adopting this approach, we guarantee that only a manageable volume of data, which can comfortably fit within the constraints of the FPGA device DRAM, is transferred at any given time, preventing any potential overflows. Additionally, the task-by-task data transfer and execution approach allowed us to seamlessly maintain compatibility with *minimap2*’s multi-threaded execution with minimal source code modifications.

#### Chaining task time predictions on hardware and software

To process chaining tasks on the FPGA-based hardware accelerator, the input chaining anchor data and the output chaining scores need to be transferred to/from the FPGA device connected to the host CPU via PCI Express interface. However, this data transfer incurs overhead, which is only justifiable if the execution time on the hardware accelerator is significantly smaller than the software execution time. Therefore, chaining tasks are processed on the FPGA-based hardware accelerator only if the total processing time (data transfer time + execution time) on hardware is smaller than the execution time on software. In this work, the hardware-software split of chaining tasks presented in our previous work^6^ was optimised with a novel approach that is based on predicted hardware/software processing times. Equation (1 and 2) depict the linear regression models formulated to predict the processing time of a given chaining task on hardware ($T_{hardware}$) and software ($T_{software}$) respectively. In these equations, *n* is the number of anchors and $K_1$, $K_2$, $C_1$, $C_2$ are constants. More details on how the equations were derived are in Supplementary Note 2.

1 $$\begin{aligned} T_{hardware} = T_{\textit{data\_transfer}}+T_{execution} = (K_1 \times n)+(II \times T_{clock} \times \textit{total\_subparts}) + C_1 \end{aligned}$$

2 $$\begin{aligned} T_{software} = K_2 \times \sum \limits _{i = 1}^{n} trip\_count_{i} + C_2 \end{aligned}$$

The linear regression models were trained by analyzing the hardware/software execution times of a set of chaining tasks measured with a representative dataset. A shell script has been provided under the *minimap2-fpga* repository to execute this one-time linear regression model training process on a different dataset if deemed necessary in future.

#### Hardware-software execution of *minimap2-fpga*

The overall hardware-software execution process of *minimap2-fpga* maintains the multi-threaded execution framework of the original *minimap2* while enhancing the chaining step through FPGA acceleration with minimal interference to the multi-threaded operation (Fig. 2b). Similar to *minimap2*, when *minimap2-fpga* initiates, multiple software threads are concurrently launched on the multi-core CPU, with each thread being assigned a batch of DNA sequence reads for processing (Fig. 2b). More details on hardware-software integration and hardware scheduling are in Supplementary Note 3.

## Conclusion

In this work, we presented *minimap2-fpga*, a hardware-accelerated version of *minimap2* that integrates an FPGA kernel optimized for chaining. *minimap2-fpga* supports mapping DNA reads from both ONT and PacBio onto a reference genome. For end-to-end execution, *minimap2-fpga* was up to 79% and 53% faster than original *minimap2* for ONT and PacBio datasets respectively, when mapping without base-level alignment. When mapping with base-level alignment, *minimap2-fpga* was up to 62% and 10% faster than *minimap2* for ONT and PacBio datasets respectively. The mapping output accuracy of *minimap2-fpga* was near-identical to that of *minimap2*. To achieve this, we had to address several challenges including scheduling overheads in multi-threaded execution and resolve an unexpected issue that affected mapping accuracy, which only became apparent during full integration. Our work highlights the challenges and importance of integrating accelerated kernels back into software workflows and offers valuable insights for other bioinformatics tools requiring heterogeneous computing accelerations.

### Supplementary Information

Supplementary Information.

## Data Availability

The experimental data used in benchmarking experiments are from publicly available datasets on NCBI Sequence Read Archive (ONT data: SRX11368472, PacBio data: PRJNA586863). Simulated reads can be created by following the instructions in Supplementary Note 4.2.
